# A survival guide for drug survival studies in dermatology: Methodological approaches and best practices

**DOI:** 10.1016/j.xjidi.2026.100548

**Published:** 2026-10-03

**Authors:** Liana Barenbrug, Linda T.H. Godding, Elke M.G.J. de Jong, Marieke M.B. Seyger, Gerjon Hannink, Juul M.P.A. van den Reek

**Affiliations:** 1 Department of Dermatology, Radboud University Medical Center (Radboudumc), Nijmegen, The Netherlands; 2 Department of Medical Imaging, Radboud University Medical Center, Nijmegen, The Netherlands

**Keywords:** Competing risk, Cox regression, Drug survival, Kaplan–Meier, Restricted mean survival time

## Abstract

The expanding therapeutic landscape for inflammatory skin diseases, combined with rising healthcare costs, has increased the need for a comprehensive method to assess and compare treatment performance in daily practice care. In this context, drug survival—the time a patient remains on a given treatment—has gained increasing attention in dermatology because it comprehensively reflects treatment effectiveness, safety, and patient and clinician preferences. However, methodological approaches and reporting practices vary considerably across drug survival studies in dermatology. In this article, we aim to provide an updated and comprehensive guide for clinical researchers on drug survival methodology, offering recommendations for performing, reporting, and interpreting drug survival analyses in a standardized manner, while highlighting best practices within dermatology. We provide guidance on fundamental concepts of drug survival analysis, including Kaplan–Meier curves, reporting standards, censoring, stratification, regression analyses, and sample size calculations. In addition, to further enhance the quality of analyses, we discuss advanced topics such as restricted mean survival time, competing risks, time-dependent effects and covariates, and meta-analyses of drug survival data. Key concepts will be illustrated using a synthetic dataset, comprising patients with psoriasis treated with biologics, complemented by references to published studies that clarify relevant methodological concepts and provide real-world dermatological examples.

## Introduction

The increasing availability of therapeutic options for inflammatory skin diseases and the rising healthcare costs have resulted in a demand for effective and comprehensive methods to evaluate treatments in daily clinical practice. In this context, the concept "drug survival" has gained prominence in the field of dermatology and has become a widely used approach in the last decade (van den Reek et al, 2015), especially in studies investigating chronic conditions such as psoriasis, atopic dermatitis, hidradenitis suppurativa, and urticaria (Ring et al, 2022; Soegiharto et al, 2024; Spekhorst et al, 2022; Thomas et al, 2024). In addition to its broad utility in dermatology, it is also frequently applied in other fields concerning immune-mediated inflammatory diseases such as rheumatological diseases (Egeberg et al, 2022; Rosenberg et al, 2023; Strunz et al, 2024) and inflammatory bowel disease (Caron et al, 2024; Kapizioni et al, 2024; Rundquist et al, 2021; Visuri et al, 2021). Note that within the literature, terms such as “drug persistence” and “drug longevity” are used alongside “drug survival.” To ensure clarity and uniformity, we consistently use the term “drug survival” throughout this manuscript.

Although the utilization of drug survival analyses is becoming more widespread, we observed significant variability in methodologies and reporting. This is notable given that drug survival studies have demonstrated their value in providing insight into the timing and reasons for treatment discontinuation (eg, lack of effectiveness, adverse events, or remission) as well as in identifying factors associated with treatment discontinuation that are relevant to clinical decision making (Phan et al, 2025; Rayner et al, 2025; Soegiharto et al, 2024; van der Gang et al, 2026). This variability emphasizes the necessity for explicit guidance on the reporting and refinement of drug survival analyses. Therefore, the aim of this article is to provide an updated, comprehensive guide on drug survival methodology to perform, report, and interpret drug survival analyses in a uniform way, while showing best practices in the field of dermatology. This will contribute to higher-quality drug survival studies and facilitate reviews and meta-analyses of drug survival data.

An overview of the concepts discussed in this article is presented in Table 1. To illustrate these concepts, a synthetic dataset, comprising patients with psoriasis undergoing treatment with biologics, will be used to enhance clarity and facilitate visualization. Information on how the synthetic dataset was created and the baseline characteristics of this illustrative population are presented in Supplementary Table S1. In addition, we refer to studies with relevant examples or clarification of methodological concepts.

**Table 1:** Overview of Drug Survival Concepts

Drug Survival Concept	Paragraph
Basic concepts of drug survival analysis	2
Defining time zero and treatment discontinuation	2.1
Cause-specific drug survival analysis	2.2
Kaplan–Meier curves	2.3
Censoring	2.4
Informative censoring	2.4.1
Stratification	2.5
Regression models in the context of drug survival	2.6
Cox regression and its assumptions	2.6.1
Variable selection	2.6.2
Unmeasured confounding and missing data	2.6.3
Exploratory versus confirmatory use of regression models	2.6.4
Sample size calculation	2.7
Advanced drug survival methodologies	3
Restricted mean survival time	3.1
Competing risks	3.2
Time-dependent effects and covariates	3.3
Meta-analysis of drug survival data	3.4
Suitable data sources	4
Artificial intelligence	5
Implications of drug survival analysis	6

## Basic Concepts of Drug Survival Analysis

Drug survival is a comprehensive outcome reflecting the effectiveness, safety, and patients’ and doctors’ preferences regarding treatment and can be defined as the time patients remain on a specific drug. Drug survival analysis is derived from traditional survival analysis, in which the time to an event is studied. In case of drug survival analyses, the event is treatment discontinuation. The probability of remaining on treatment can be estimated for specific time points (Bland and Altman, 1998).

### Defining time zero and treatment discontinuation

For drug survival analyses, it is crucial to clearly report both start and end of follow-up. The definition of time zero may vary depending on the data source used (eg, prescription date, dispensing date, or patient or physician treatment initiation date), each reflecting a different clinical context and potentially resulting in different follow-up periods. Similarly, the data source may influence how treatment discontinuation is defined. In real-world dermatology cohorts, permanent treatment discontinuations may occur, but treatment switches or temporary interruptions are often more common. Whether a temporary treatment interruption is classified as discontinuation may vary between studies and may also depend on whether such interruptions are captured in the underlying dataset. In addition, the definition of treatment discontinuation is challenged by differences in dosing frequency between therapies. For instance, long-acting biologics and particularly the anticipated introduction of extended half-life biologics will challenge us to redefine the treatment discontinuation event in a manner that accounts for clinically meaningful gaps between consecutive drug administrations (Yilmaz and Torres, 2024). Moreover, the growing use of individualized treatment approaches, including interval extension and on-demand treatment, challenges traditional discontinuation definitions that were developed for fixed dosing regimens (van Riel et al, 2024). Therefore, although standardized definitions would improve comparability across drug survival studies, a single definition may not be feasible given heterogeneity in data sources and their ability to capture treatment patterns. Consequently, studies should explicitly report how time zero and treatment discontinuation were defined.

### Cause-specific drug survival analysis

Drug survival can be analyzed as an overall measure, often referred to as “overall drug survival,” in which all reasons for treatment discontinuation are treated as a single event. In such analyses, it can be difficult to interpret whether higher or lower drug survival estimates are favorable or unfavorable, especially when positive outcomes (ie, discontinuation due to remission) and negative outcomes (ie, discontinuation due to ineffectiveness or adverse events) are combined as a single event. In addition, it is important to know whether or not the reason for discontinuation might be related to the treatment (ie, ineffectiveness, adverse events, or remission). Therefore, it is strongly recommended to complement overall drug survival with cause-specific analyses that distinguish between different reasons of discontinuation. This ensures a more informative and interpretable assessment of drug performance (Schlosser et al, 2025; Soegiharto et al, 2024).

### Kaplan–Meier curves

Survival probabilities can be visualized with Kaplan–Meier curves (Figure 1). The occurrence of the event of interest, in this case “drug discontinuation at any timepoint,” results in a decrement in the Kaplan–Meier survival curve at that point. Each such event as well as censoring (see also section on censoring) reduces the number of “patients at risk,” that is, individuals still under observation and event free at each time point. When interpreting Kaplan–Meier curves, it is important to recognize that once only a small number of patients remain at risk (ie, many individuals experienced the event or were censored), the survival curve may exhibit large downward steps, limiting the reliability of estimates in the tail of the curve. To support appropriate interpretation, it is recommended to include 95% confidence intervals (CIs) and the number of patients at risk at each time point (Figure 1, sections 1 and 2). Although the survival probability at a certain clinically relevant time point can be extracted from the curve, it is also recommended to explicitly report this estimate including 95% CI in the manuscript. For example, on the basis of Figure 1, the 5-year overall drug survival of biologics in patients with psoriasis is 46.3% (95% CI = 43.4–49.5%) (Figure 1, section 3).

**Figure 1:**
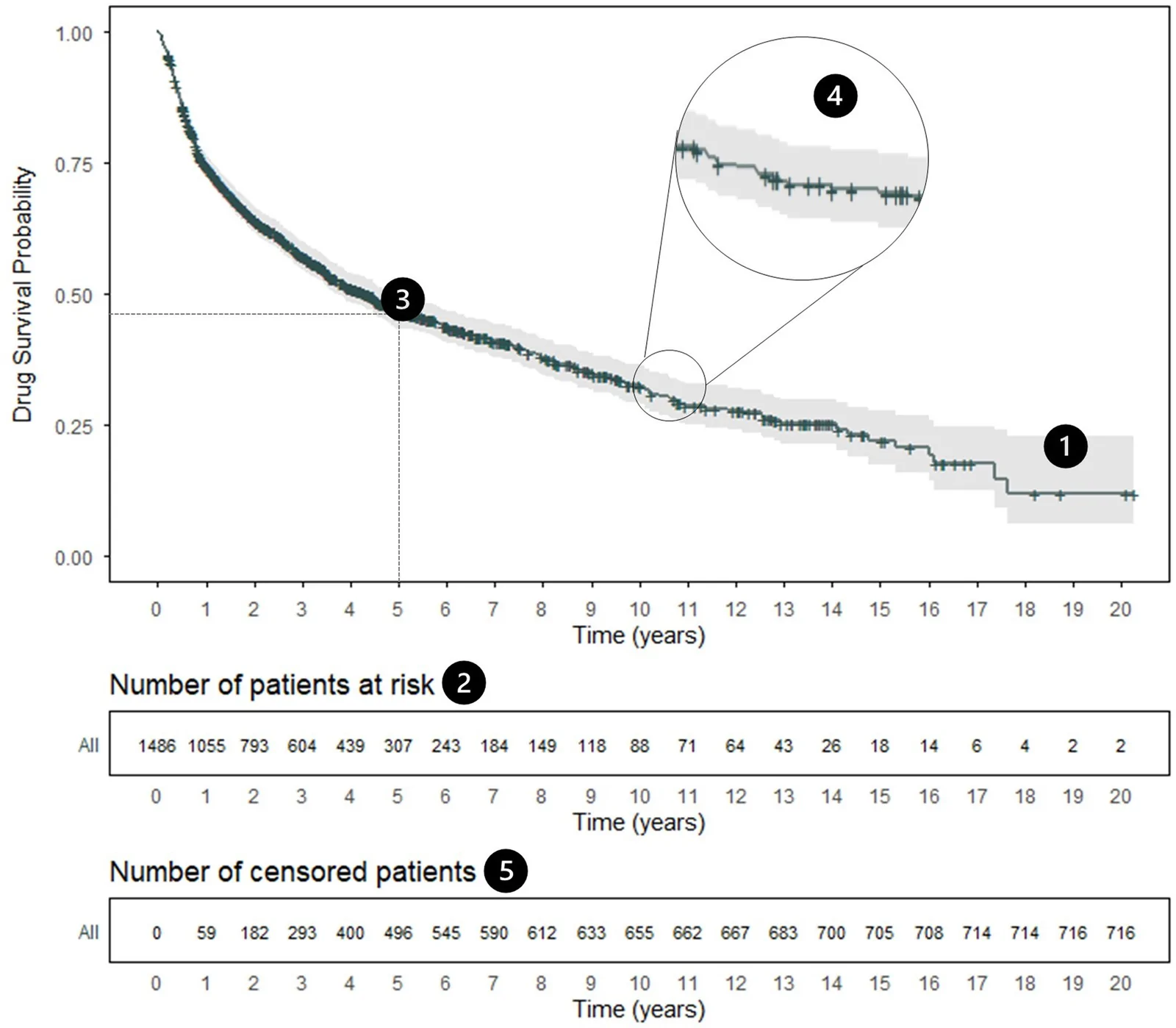
**Recommendations for reporting Kaplan–Meier curves.** Shown is a Kaplan–Meier curve showing overall drug survival of biologics in patients with psoriasis. To support the appropriate interpretation of Kaplan–Meier curves, it is recommended that the following be presented: 1. confidence intervals, 2. a table with the number of patients at risk at each time point, 3. the survival estimate of a clinically relevant time point alongside the curve, 4. symbols that visualize censoring, and 5. a table with the number of censored patients to indicate the time points at which censoring predominantly occurs. Data from synthetic dataset.

### Censoring

If a patient’s follow-up duration is shorter than the prespecified time frame of the survival analysis and no event has occurred by the end of follow-up, the patient is considered censored. In such cases, we only know that the patient remained event free up to their last observed follow-up time, and only the information available up to that time is included in the analysis. When the analysis is focused on a specific event of interest, such as a particular reason for drug discontinuation, other reasons for discontinuation may be treated as censored observations in a cause-specific survival analysis. For example, if the event of interest is discontinuation due to adverse events, discontinuation due to ineffectiveness, pregnancy, death, or other causes would be censored at the time they occur (note that in some cases, a competing risk analysis might be more appropriate, see also section on competing risks). Censoring is often visualized by a tick mark or other symbol in the curve (Figure 1, section 4). When a large number of patients are censored, the tick marks denoting censored observations may be difficult to distinguish. Reporting the number of censored patients would help clarify this and could indicate the time points at which censoring predominantly occurs (Figure 1, section 5).

#### Informative censoring

Kaplan–Meier analyses rely on the assumption that the reason for censoring is unrelated to the event of interest, which is referred to as “noninformative censoring” (Schober and Vetter, 2018). In practical terms, this means that patients who are censored should not have a systematically different subsequent risk of treatment discontinuation from patients who remain under observation, after accounting for relevant observed characteristics. This assumption may, however, not consistently hold in real-world dermatology studies. For example, patients with an inadequate treatment response may be more likely to transfer to another (academic) hospital or change healthcare provider and, consequently, will be censored as they become lost to follow-up. Because inadequate treatment response is also associated with an increased likelihood of treatment discontinuation, the risk of discontinuation among censored patients may differ from that among patients who remain under observation. In this situation, censoring is informative and may result in biased drug survival estimates.

When informative censoring is suspected, for example, on the basis of timing of censoring, investigators should first investigate predictors of loss to follow-up and assess whether these predictors are also associated with treatment discontinuation. One commonly used approach is inverse probability of censoring weighting (IPCW) (Willems et al, 2018). In this approach, the probability of remaining uncensored is estimated from observed patient characteristics, and patients are weighted inversely to this probability. Therefore, individuals who are less likely to remain under observation contribute more information to the analysis, aiming to create a weighted population in which censoring is conditionally independent of the outcome. IPCW can be incorporated into survival or regression analyses. However, IPCW cannot fully correct for informative censoring caused by unmeasured factors. Its validity depends on adequate measurement and modeling of variables associated with both censoring and the outcome. Investigators should therefore report the extent and potential determinants of loss to follow-up, consider stabilized weights and appropriate diagnostics, and perform sensitivity analyses when the assumption of noninformative censoring is uncertain. Recent methodological work also emphasizes the importance of appropriate variable inclusion and assessment of extreme weights when applying censoring or observation-process weighting (Tompkins et al, 2025).

### Stratification

Stratification can be used when there is an interest in specific characteristics of the population that might influence treatment discontinuation. For example, patients initiating their first biologic therapy typically represent a clinically distinct population from those who have previously failed 1 or more biologics. Because prior biologic exposure is strongly associated with subsequent treatment persistence, stratified drug survival analyses may provide more clinically meaningful estimates (Figure 2a) (Lazaridou et al, 2024). Similarly, stratification by sex may be informative. Males and females may differ in disease burden, treatment expectations, and reasons for treatment discontinuation (Gonzalez-Cantero et al, 2023). Consequently, drug survival observed in the overall population may not adequately reflect drug survival patterns within each sex (Figure 2b) (Barenbrug et al, 2025). Survival curves from stratified analyses can be compared using the nonparametric log-rank test (Mantel–Cox test) to determine whether there are statistically significant differences between groups. When survival curves cross, one treatment may appear better early on, and the other may appear better later, suggesting that the treatment effect is not constant over time. However, curves can also cross by chance, particularly when few patients remain at risk late in follow-up, and crossing alone does not automatically invalidate standard analyses (Dormuth et al, 2022). Rather than serving as a decision rule, crossing curves should prompt a closer examination of when benefits or harms occur and whether this time pattern is clinically plausible. In such cases, alternative approaches, such as weighted log-rank tests with clear clinical justification or restricted mean survival time (RMST) (see also the sections on Cox regression and its assumptions and RMST), which provides an interpretable summary of average survival up to a clinically meaningful time point, may be considered.

**Figure 2:**
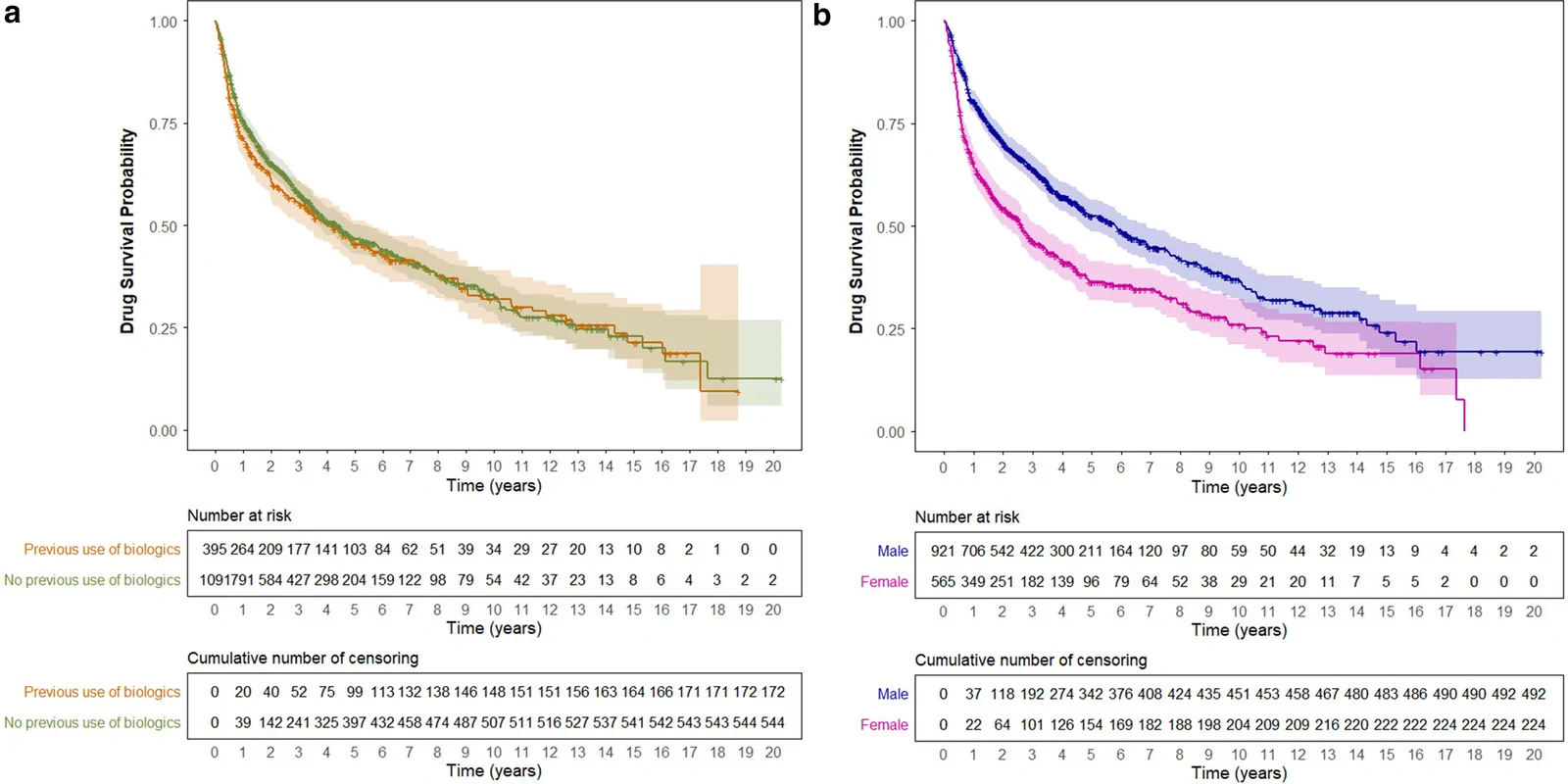
**Kaplan–Meier curve showing overall drug survival of biologics in patients with psoriasis.** (**a**) Stratified for prior use of biologics. (**b**) Stratified for sex. Data from synthetic dataset.

### Regression models in the context of drug survival

Regression models can be used to examine the relationship between a given variable and drug discontinuation while adjusting for relevant covariates. Currently, the most commonly used regression model is the Cox proportional hazard model, which yield hazard ratios (HRs) that describe the relative instantaneous hazard of treatment discontinuation associated with a covariate, conditional on the other variables included in the model. The remainder of this section will focus on this model in more detail, highlighting key considerations for its application and interpretation. However, no single regression model is appropriate for all drug survival analyses; the choice should be guided by the research question, the structure of the data, and the assumptions underlying each model.

#### Cox regression and its assumptions

The Cox proportional hazard model relies on 3 assumptions that should be assessed. First, HRs should remain constant throughout the period under study (proportional hazard assumption). However, this assumption may be questionable in dermatological drug survival studies. For example, adverse events may lead to early discontinuation, whereas treatment ineffectiveness may become more relevant after longer treatment exposure. Consequently, the relative hazard of discontinuation may change over time. When the proportional hazards assumption is violated, several approaches are available. In settings where the proportional hazards assumption is violated for a stratification variable, stratified Cox models can be used, although the effect of that variable can no longer be estimated directly (Kleinbaum, 1996). Alternatively, time-dependent covariates or time-varying coefficients can be incorporated to allow associations with discontinuation to change over time (section on time-dependent effects and covariates provides more details). In settings where the proportional hazards assumption is clearly violated, reporting time-specific effects or RMST (section on RMST) may provide a more interpretable alternative.

Second, the survival times of different individuals in the study population should be independent. This assumption may be violated when patients are clustered within the same treatment center or physician because they may have similar treatment strategies, patient characteristics, or follow-up patterns. When relevant clustering is present, this should be accounted for using approaches such as robust standard errors or multilevel (frailty) Cox models.

Third, censoring should be uninformative, which is previously discussed in informative censoring).

Note that when the assumptions of the Cox proportional hazard model are violated, flexible parametric models can also be considered and should be selected on the basis of the research question and supported by appropriate model-fit assessment (Motedayen Aval et al, 2025; Royston and Lambert, 2014; Yiu et al, 2020). Importantly, violation of the proportional hazards assumption alone does not require a parametric model.

#### Variable selection

Appropriate adjustment for confounding in observational drug survival studies depends on careful selection of variables. Although univariable variable selection, that is including those independent variables in a multivariable model that show significant association with the outcome in univariable models, is one of the most popular approaches in many fields of research, it should be generally avoided (Heinze et al, 2018; van Diepen et al, 2017). Statistical significance in a univariable analysis does not determine whether a variable is an important confounder, and clinically important confounders may be excluded because of limited statistical power. Conversely, variables may appear associated with discontinuation because of incorrect adjustment for confounding or chance. Therefore, variable selection should preferably be guided by the research question; subject-matter knowledge; previous evidence; and, where appropriate, a prespecified causal framework such as a directed acyclic graph (DAG) rather than solely by univariable *P*-values (Greenland et al, 1999; Shrier and Platt, 2008). Figure 3 shows an illustrative DAG for the relationship between body mass index and drug discontinuation due to adverse events. This DAG indicates that age, sex, and smoking are confounding variables because they are associated with both the exposure and the outcome. The use of a DAG facilitates the identification of confounders and helps avoid inappropriate adjustment for other variables (eg, mediators, variables lying on the causal pathway between the exposure and the outcome, or colliders, events that are caused by both the exposure and the outcome). Another important consideration is the presence of effect modification, for which interaction terms should be integrated within the regression model. In addition to conventional regression-based approaches, methods for addressing confounding, such as propensity score–based methods and inverse probability weighting, can also be considered (Desai and Franklin, 2019).

**Figure 3:**
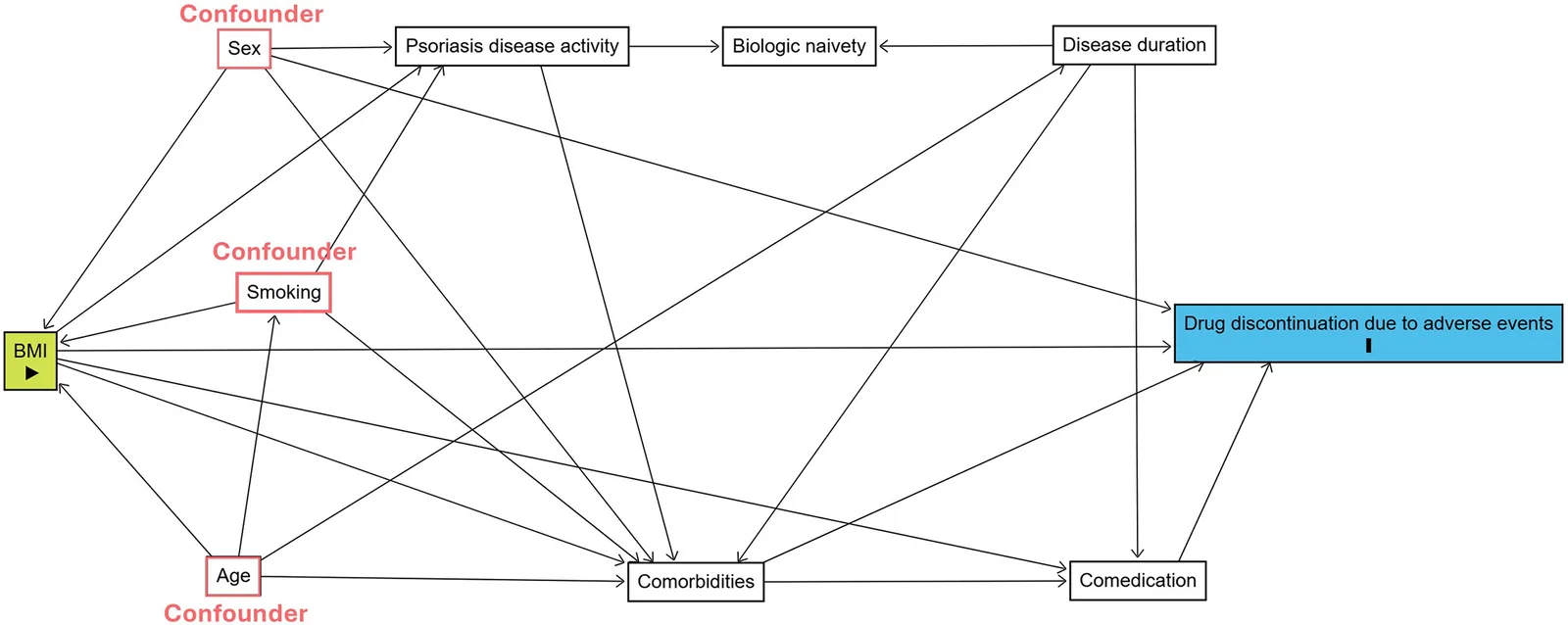
**DAG for the relationship between BMI (exposure) and drug discontinuation due to adverse events (outcome).** Confounder: A variable that is associated with both the exposure and the outcome. BMI, body mass index; DAG, directed acyclic graph.

#### Unmeasured confounding and missing data

Drug survival studies are also vulnerable to residual and unmeasured confounding. Important determinants of treatment discontinuation may not be adequately captured in routinely collected dermatological datasets. These may include disease severity not fully reflected by recorded clinical scores, physician preference, patient preferences, adherence, treatment expectations, insurance restrictions, reimbursement policies, drug availability, and changes in treatment access over (calendar) time. For example, apparent differences in drug survival between two treatments may partly reflect differences in reimbursement or prescribing restrictions rather than differences in treatment effectiveness or tolerability. Multivariable adjustment can only address confounding by variables that are measured with sufficient accuracy. Therefore, residual confounding may remain owing to unmeasured or even unknown factors. Investigators should discuss plausible unmeasured confounders and consider sensitivity analyses to assess the robustness of the observed association to unmeasured confounding (Haneuse et al, 2019).

Missing data are a common challenge in regression analyses of real-world drug survival data and may introduce bias if handled inappropriately (van Buuren, 2018). Therefore, the extent and pattern of missing data should be assessed, and the potential missingness mechanism, that is, missing completely at random (MCAR), missing at random (MAR), or missing not at random (MNAR), should be considered when selecting an appropriate approach for handling missing data (Little and Rubin, 2002). Dependent on the missingness mechanism, there are several methods available to handle missing data. One example is multiple imputation, in which the missing values are imputed with multiple plausible values (Austin et al, 2021). This is also often used in drug survival studies within the dermatological field (Spekhorst et al, 2022).

#### Exploratory versus confirmatory use of regression models

Cox regression can be used in both exploratory and confirmatory analysis. Often, they are used in an exploratory manner, to identify which variables, such as patient characteristics or treatment-related factors, may influence the observed drug survival outcomes. In such studies, variables that are thought to potentially affect drug survival are typically added directly to the model, without a prespecified primary independent variable or a predefined set of variables for statistical adjustment. Because all variables are included simultaneously, each variable can act as a confounder, mediator, or collider for the others, which leads to estimates that are difficult to interpret. Therefore, the estimates should be interpreted carefully and cannot necessarily be defined as predictors (Lewer et al, 2025). In regression analyses, each coefficient represents the association between that variable and the outcome, conditional on all other variables in the model. The coefficient for the main exposure of interest reflects its effect after adjusting for potential confounders, which is usually the primary focus of reporting. Coefficients for covariates included solely for adjustment (eg, age, sex, comorbidity scores) describe their conditional associations with the outcome, but these are often not clinically meaningful and can be misleading if interpreted as causal effects. Therefore, it is generally recommended to report only the estimates for the main exposure variable (Westreich and Greenland, 2013). When the analysis is confirmatory, the model specification, covariates, and primary hypotheses should ideally be defined a priori (see the section on variable selection).

### Sample size calculation

In drug survival analyses, it is common practice to compare two or more drugs or to stratify by patient characteristics to demonstrate differences between patient groups. Power and sample size calculations can ensure that the study population is sufficiently large to detect clinically meaningful differences between 2 groups. Sample size calculations for drug survival studies should not be restricted to a comparison of 2 groups with a binary endpoint but should account for the time-to-event nature of the outcome.

For drug survival studies, investigators should consider the expected number of treatment discontinuations, the number of treatment groups, the number and type of covariates, competing risks, anticipated censoring, and the effect size of clinical interest (Zurakowski and Staffa, 2023). Simulation-based approaches can be particularly useful when these features are complex or when the planned analysis involves time-varying effects, competing risks, or RMST (Austin, 2012; Latouche and Porcher, 2007; Luo et al, 2019).

For rare or ultra-rare diseases, conventional sample size requirements may be difficult or impossible to achieve. In such cases, Bayesian approaches, such as the dynamic borrowing approach, may be particularly relevant, because they incorporate relevant information from previous trials, registries, or other external data sources, thereby reducing the amount of new information required to achieve a prespecified level of precision or decision certainty (Berry et al, 2010b). Additional approaches to improve efficiency in small populations include adaptive or group-sequential designs, enrichment strategies, shared control groups in multiarm studies, and careful adjustment for strong prognostic covariates (Berry et al, 2010b; Kidwell et al, 2022). In rare diseases, these approaches should be considered as ways of increasing statistical efficiency rather than as methods that universally eliminate the need for adequate sample size. Importantly, the choice of method should be driven by the estimand (ie, the precise treatment effect that a study aims to estimate and which reflects the clinical question of interest), and simulation-based sample size or power calculations are often preferable when standard formulas do not adequately reflect the planned analysis (Kahan et al, 2024). Note that when sample size requirements become so large that achieving sufficient statistical power may not be feasible, this does not necessarily diminish the value of the study. Because rare adverse events are also often not detectable in randomized clinical trials, it is recommended to conduct multiple observational studies with comparable methodologies and subsequently pool their results in a meta-analysis (Hernán, 2022). This approach can provide more precise and reliable estimates.

## Advanced Drug Survival Methodologies

Although conventional drug survival methods are widely used to evaluate and compare treatment performance, they may not always be sufficient to address more complex clinical and methodological challenges. The following section discusses the methodological approaches that have been developed to address some of these challenges, highlighting their rationale, applications, and added value in drug survival research.

### RMST

The RMST is a valuable alternative to conventional methods in drug survival studies when the proportional hazards assumption is violated (Han and Jung, 2022; Royston and Parmar, 2011). This can be, for example, the case in studies comparing multiple treatments with distinct mechanisms of action and different temporal discontinuation profiles, potentially resulting in crossing drug survival curves. In addition, it is a valuable complement to conventional methods when clinically interpretable measures of drug survival are desired.

Conceptually, RMST represents the area under the drug survival curve up to a designated time point (Figure 4). It serves as the average drug survival time within a prespecified period, taking censoring into account. This time horizon should be specified a priori and chosen on the basis of clinical relevance. Although selecting an appropriate time point can be challenging, a clear clinical and methodological rationale is essential, because the estimated difference in RMST and its statistical significance depend on this chosen time horizon. Differences in drug survival between groups, for example, between males and females, can then be quantified as the difference in RMSTs (Figure 4c). It is also possible to estimate RMSTs adjusted for covariates (Tian et al, 2014; Uno et al, 2014).

**Figure 4:**
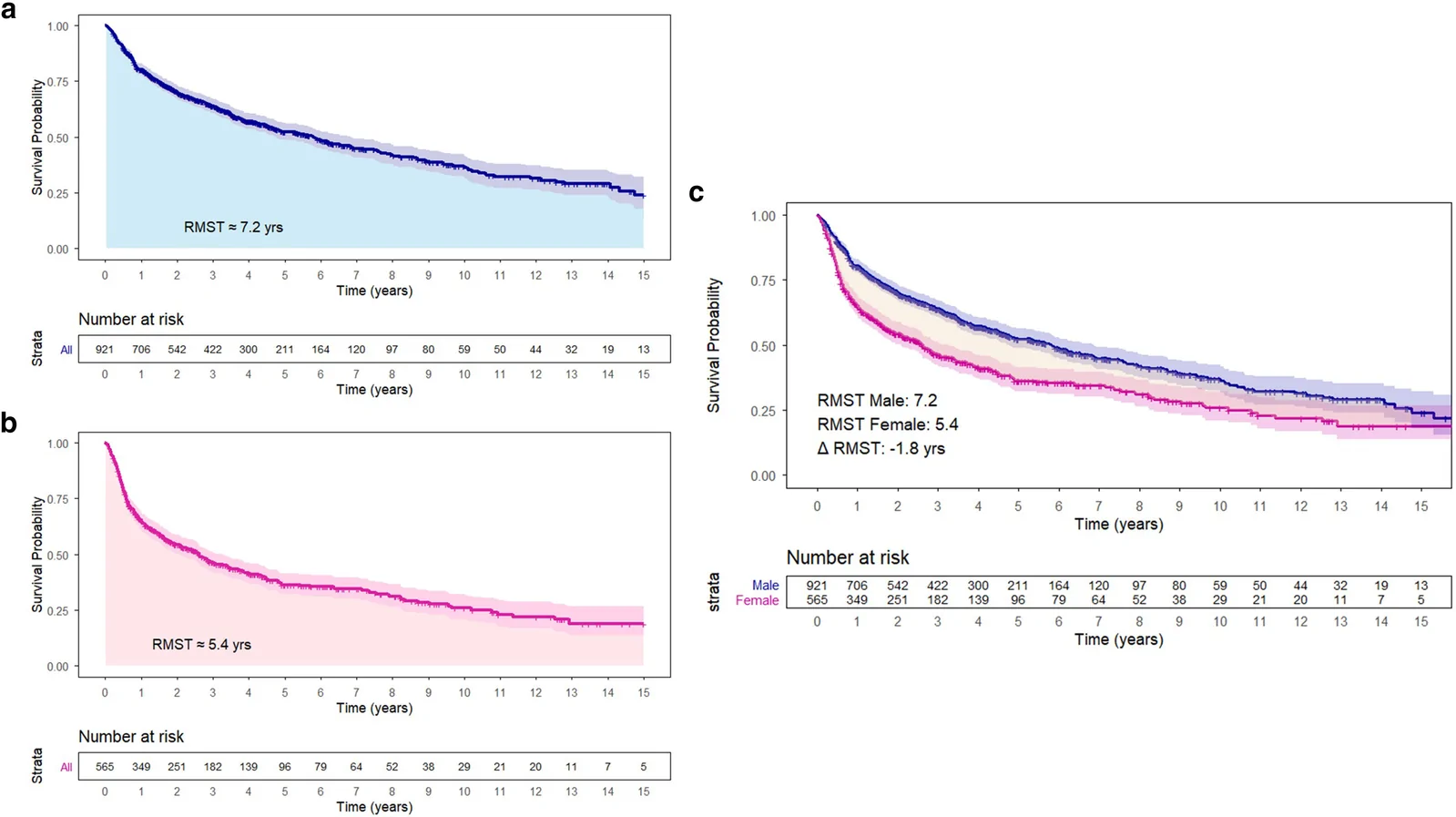
**RMST of biologics in patients with psoriasis at 15 years.** (**a**) For males. (**b**) For females. (**c**) Difference in RMST between males and females. RMST male 7.2 (95% CI = 6.7–7.7): On average, male patients stay on the biologic 7.2 years of the first 15 years of treatment. RMST female 5.4 (95% CI = 4.8–6.0): On average, female patients stay on the biologic 5.4 years of the first 15 years of treatment. ΔRMST = −1.8 (95% CI = −2.5 to −1.0): Female patients stay on average 1.8 years shorter on biologics over the first 15 years of treatment compared with males. Data from synthetic dataset. CI, confidence interval; RMST, restricted mean survival time.

Although RMST has not yet been widely adopted in the field of dermatology, its application has recently been demonstrated in a study investigating the drug survival of IL-17 and IL-23 inhibitors compared with those of other biologics for psoriasis, in which RMST was applied to deal with nonproportional hazards (Motedayen Aval et al, 2025). Another study (Alabas et al, 2023) used the RMST to quantify the difference in mean drug survival time between methotrexate and adalimumab. In both studies, the specified time horizon was set at 2 years, primarily on the basis of the median and mean duration of follow-up.

### Competing risks

The Kaplan–Meier method estimates the probability of a single event occurring over time. When other events occur that make the event of interest impossible, that is, competing events/risks, Kaplan–Meier does not account for them correctly. Competing risks can be defined as any event that hinders the occurrence of the event of interest or modifies the chance that the event of interest occurs (Ranstam, 2018; Sayers et al, 2018). For instance, in the context of cause-specific drug survival analyses where treatment discontinuation due to adverse events is the event of interest, competing events may include other reasons for discontinuation, such as, for example, ineffectiveness, remission, or pregnancy (Choi et al, 2018; Paris et al, 2025; Phan et al, 2025). Specifically for dermatology, this may be the case for Jak inhibitors, where the proportion of patients discontinuing treatment due to adverse events may be comparable to that discontinuing due to ineffectiveness (van der Gang et al, 2026). The Kaplan–Meier method treats patients who experience a competing event as censored and therefore implicitly assumes that they remain at risk for the event of interest, even though they can no longer experience the event of interest. This leads to an overestimation of the true probability of the event of interest. It is discussed that competing risk modeling should be considered when either (i) the proportion of patients experiencing the competing event is equal to or exceeds that of the event of interest or (ii) the absolute proportion of competing events is greater than 10% (Austin et al, 2016; Berry et al, 2010a). In general, the greater the occurrence of the competing event, the greater the bias is when competing events are treated as censored.

To properly account for competing risks, the cumulative incidence function (CIF) can be used. This method denotes the probability of discontinuation due to the event of interest before a specific time and before the occurrence of another event. In case of adverse events as event of interest, this means that patients who, for example, discontinue their treatment due to ineffectiveness, are considered no longer at risk for discontinuation due to adverse events (Schuster et al, 2020).

Considering the example of cause-specific drug survival analysis where discontinuation due to adverse events is the event of interest, when applying a traditional Kaplan–Meier survival method, all other reasons for discontinuation are treated as censored observations (Figure 5a). When we would like to treat discontinuation due to ineffectiveness as competing event, we can use the CIF. In Figure 5b, the CIF for discontinuation due to adverse events is shown in red. At 10 years, the CIF shows a cumulative incidence of 0.19 (19.0%). In contrast, the Kaplan–Meier curve in Figure 5a estimates the survival probability for discontinuation due to adverse events at 0.73 (1–0.73 = 0.27 = 27.0%). This discrepancy demonstrates that the Kaplan–Meier method tends to overestimate the probability of discontinuation due to adverse events by failing to account for discontinuation due to ineffectiveness. When competing risks are considered in the analyses, the Cox proportional hazard model is no longer appropriate. Depending on the research question, the Fine and Gray subdistribution hazard model can be used for prognostic research and the cause-specific hazard model is suitable for etiologic research (Andersen et al, 2012).

**Figure 5:**
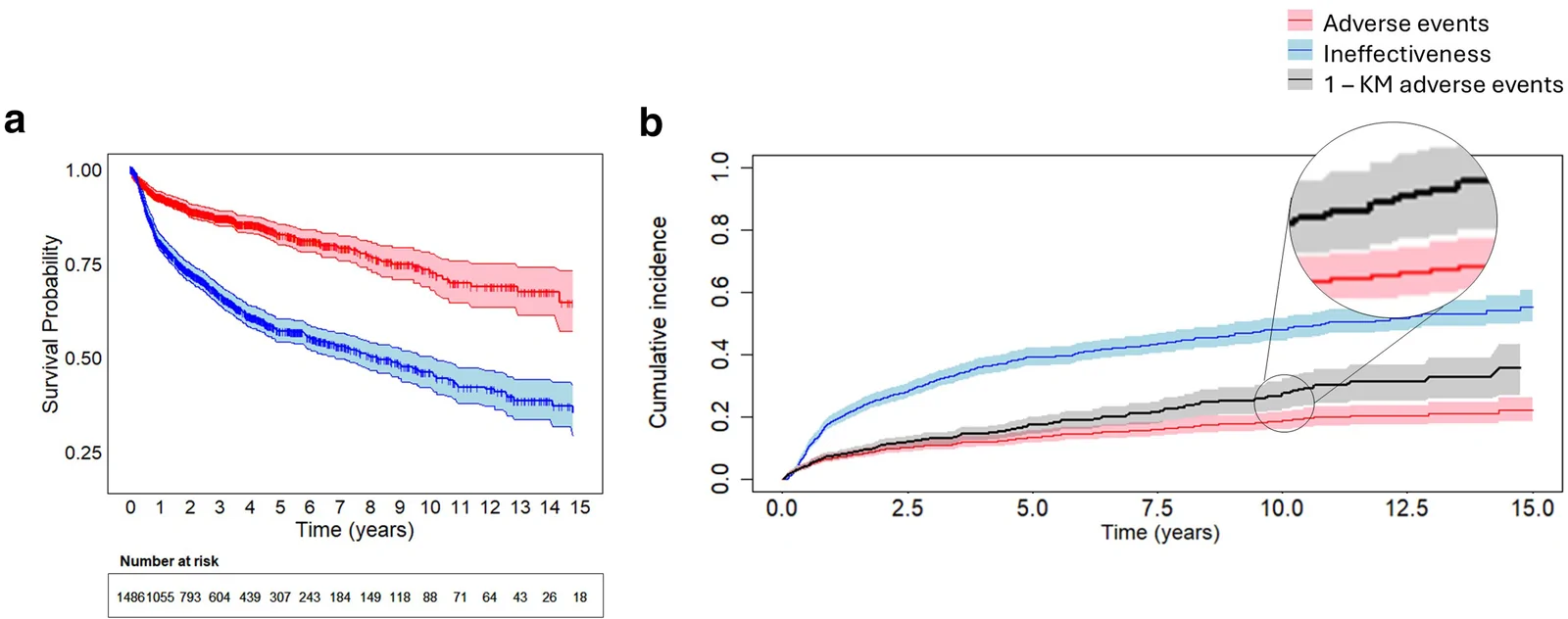
**Handling competing risks in drug survival analyses.** (**a**) Traditional Kaplan–Meier curves according to reason for discontinuation. (**b**) CIFs for discontinuation due to adverse events (red), discontinuation due to ineffectiveness (blue), and the 1-Kaplan–Meier curve for discontinuation due to adverse events (black). To illustrate the impact of competing risks, the CIF can be compared with 1 − Kaplan–Meier. Whereas 1 − KM ignores competing events and treats patients experiencing them as still at risk, the CIF accounts for competing events and therefore provides the true probability of the event of interest. Comparing the 2 curves highlights how Kaplan–Meier can overestimate the risk when competing risks are present. Data from synthetic dataset. CIF, cumulative incidence function.

Because highly effective targeted therapies increasingly enable sustained disease control or remission, treatment discontinuation due to these reasons is expected to become more common. This is already evident in chronic urticaria, where remission is the primary reason for omalizumab (Litovsky et al, 2023; Soegiharto et al, 2024). Consequently, discontinuation due to remission is likely to become an increasingly important competing event in cause-specific drug survival analyses. To date, studies applying competing risk analyses within the field of dermatology are exceptionally scarce. To our knowledge, one of the few published examples is a retrospective multicentric French study (Litovsky et al, 2023) on omalizumab drug survival in patients with chronic urticaria. In this study, the authors dealt with the presence of competing risks by showing the 1 minus CIF and by applying the Fine and Gray subdistribution hazard model.

### Time-dependent effects and covariates

In adjusted drug survival analyses, covariates are often measured only at baseline and considered to be fixed throughout the follow-up period. However, in certain cases, the effect of a baseline covariate becomes stronger or weaker over the course of treatment, or the covariate itself changes over time (Dekker et al, 2008; Zhang et al, 2018). To test whether a baseline covariate has different effects over time, HRs can be calculated for different time periods within one Cox model. When the covariate itself changes over time, this covariate can be added to the Cox model as time-dependent covariate. A treatment episode is then divided into time periods corresponding to changes in the risk factor. For instance, previous studies in dermatology applied time-dependent covariates for periods during which comedication was used, psoriatic arthritis was present as comorbidity, or disease activity changed (Iskandar et al, 2018; May et al, 2025; Motedayen Aval et al, 2025). Note that when incorporating time-dependent covariates, assessing the proportional hazards assumption remains critical.

### Meta-analysis of drug survival data

Meta-analyses compile evidence from multiple studies, enhancing statistical power, reliability, and generalizability of findings. There are several approaches to perform a meta-analysis on drug survival data, each having its own strengths and limitations (Combescure, 2026). One possibility is to pool survival estimates at specific time points (Lin et al, 2018; No et al, 2018). However, this approach has several limitations: it does not account for variation in risk over time, its relevance depends on the selection of clinically relevant time points, and repeating the estimation across multiple time points is not recommended because within-study correlations are ignored and survival probabilities may increase over time. Another approach is to estimate pooled HRs at specific time points using data extracted from Kaplan–Meier curves presented in the included studies. This method allows single-arm studies to be incorporated into pairwise comparisons, for example, when comparing two biologics (Mourad and Gniadecki, 2020). A limitation, however, is again that variation in risk over time cannot be assessed and that its relevance depends on the selection of clinically relevant time points. To overcome this, Kaplan–Meier curves can be used to calculate HRs with CIs, which can be visualized in a summary survival curve (Pham et al, 2024; Thomas et al, 2024). Advantages of this approach include the ability to incorporate single-arm studies and that the models account for different numbers of patients and follow-up time between studies. For example, survival estimates at a given time point also incorporate data from studies that ended before that time point, it is essential to recognize that studies with shorter follow-up—for example, those reporting survival only up to 1 year—may still influence the summary curve beyond their observed follow-up period.

Overall, although each meta-analytic approach to drug survival data offers distinct advantages, careful consideration of their underlying assumptions and limitations is essential to ensure that the pooled estimates are both clinically meaningful and methodologically sound.

## Suitable Data Sources

For accurate interpretation of drug survival analyses, selecting the right data source is essential. Data can be obtained, for example, from registries, electronic health records, insurance claims, or pharmacy records. Among these, registry and electronic health record data are the most informative and thus most recommended for drug survival analyses. Although claims and pharmacy data often cover a large patient population, they have notable limitations: (i) actual medication use cannot be confirmed at a physician or patient level; (ii) missing information on the date and cause of discontinuation; (iii) non–drug-related events, such as insurance changes, relocation, and family planning, cannot be distinguished from drug-related issues; and (iv) claims data may not represent the entire population because insurances could vary significantly between countries and does not reflect the uninsured population. Consequently, previous research demonstrated lower drug survival estimates when pharmacy or claims data are utilized than when registry or electronic health record data are used (Thomas et al, 2024). Therefore, it is important to consider the data source used when interpreting drug survival data.

## Artificial Intelligence

Artificial intelligence (AI) and machine learning may increasingly contribute to drug survival research, particularly as real-world dermatology datasets become larger and more complex. For example, AI-based natural language processing may further facilitate the extraction of treatment discontinuation and its reasons from electronic patient records, which are often incompletely captured in structured databases (Paganelli et al, 2024). In addition, machine-learning approaches can be used to predict treatment discontinuation on the basis of large numbers of baseline and longitudinal variables (Du et al, 2023; Meng et al, 2023). More advanced machine-learning survival models may also allow flexible modeling of nonlinear associations, interactions, longitudinal covariates, and heterogeneous treatment effects, including time-varying and covariate-dependent treatment effects using electronic health record data (Bica et al, 2021). However, AI should be regarded as a complementary tool rather than a replacement for appropriate drug survival methodology. Prediction and causal inference address different questions, and a highly predictive model does not necessarily provide an unbiased estimate of treatment effects. Moreover, complex models require appropriate internal and external validation and may be difficult to interpret in clinical practice.

## Implications of Drug Survival Analysis

Drug survival is a comprehensive indicator of treatment effectiveness, safety, tolerability, and patient and physician preference, which makes the method suitable for evaluating treatments for chronic diseases. Drug survival analyses can be applied when the performance of drugs must be evaluated on its own or when different drugs or populations must be compared.

In this article, we summarize the fundamental concepts of drug survival analyses and present advanced methodological approaches to enable more robust and informative analyses. In conclusion, this work aims to enhance the methodological rigor and consistency of future drug survival studies.

## Data Availability Statement

The data of the synthetic dataset will be shared on reasonable request to the corresponding author.

## ORCIDs

Liana Barenbrug: http://orcid.org/0000-0003-2405-4233

Linda T. H. Godding: http://orcid.org/0009-0008-4490-8623

Elke M. G. J. de Jong: http://orcid.org/0000-0003-3872-5704

Marieke M. B. Seyger: http://orcid.org/0000-0003-0516-8294

Gerjon Hannink: http://orcid.org/0000-0001-9526-3775

Juul M. P. A. van den Reek: http://orcid.org/0000-0002-3642-2673

## Conflict of Interest

LTHG received a speaking fee from Almirall, LEO Pharma, Eli Lilly, and Sanofi. The funding is not personal but goes directly to the Department of Dermatology, Radboud University Medical Center (Radboudumc) (Nijmegen, The Netherlands). EMGJdJ received research grants for the Department of Dermatology of the Radboud University Medical Center from AbbVie, Almirall, J&J, Leo Pharma, Lilly, and UCB and acted as consultant and/or paid speaker for and/or participated in research sponsored by companies that manufacture drugs used for the treatment of psoriasis, including AbbVie, Almirall, Boehringer-Ingelheim, Bristol Myers Squibb, J&J, Leo Pharma, Lilly, and UCB. All funding is not personal but goes to the Institution Radboud University Medical Center. MMBS received grants from/was involved in clinical trials from Eli Lilly and Janssen. She served as a consultant for BMS, Eli Lilly, Janssen, and Novartis; fees were paid directly to the institution. JMPAvdR carried out clinical trials for AbbVie, Celgene, Almirall, Astrazeneca, and Janssen and has received speaking fees/attended advisory boards from AbbVie, Janssen, Zoetis, BMS, Almirall, LEO Pharma, Novartis, UCB, and Lilly and reimbursement for attending or chairing a symposium from Janssen, Pfizer, Celgene, AbbVie, and EADV. JMPAvdR is coordinator of the BioCAPTURE registry for which the institution has received funding from Abbvie, Janssen, Lilly, Almirall, Novartis, UCB, and LEO Pharma. All funding is not personal but goes to the independent research fund of the Department of Dermatology of Radboudumc. The remaining authors state no conflict of interest.
