# Supplementary material for: A survival guide for drug survival studies in dermatology: Methodological approaches and best practices
Source: JID Innov. 2026 Oct 3;7(1):100548. doi: 10.1016/j.xjidi.2026.100548 (PMC13756787; doi:10.1016/j.xjidi.2026.100548)
Supplement: Supplementary file 1 [file mmc1.pdf]

SUPPLEMENTAL MATERIALS

Supplemental Table S1| Baseline characteristics of patients with psoriasis (synthetic dataset)

	**N = 1,486**
**Sex**	
Males	921 (62.0%)
Females	565 (38.0%)
**Age at onset (years)**	25 (17, 37)
**Age at start biologic (years)**	49 (38, 59)
**Disease duration (years)**	19 (11, 27)
**BMI (kg/m^2)**	27.6 (26.0, 30.0)
**Smoking (yes)**	359 (24.2%)
**Baseline PASI**	10 (8, 14)
**Psoriatic arthritis**	
Yes	406 (27.0%)
No	1,080 (73.0%)
**Family history of psoriasis**	
Yes	954 (64.0%)
No	532 (36.0%)
**Biologic**	
**TNFα-inhibitors**	
Etanercept	257 (17%)
Adalimumab	603 (41%)
Certolizumab	18 (1.2%)
**IL12/23-inhibitor**	
Ustekinumab	368 (25%)
**IL17-inhibitors**	
Secukinumab	47 (3.2%)
Ixekizumab	66 (4.4%)
Brodalumab	16 (1.1%)
Bimekizumab	14 (0.9%)
**IL23-inhibitors**	
Guselkumab	39 (2.6%)
Risankizumab	46 (3.1%)
Tildrakizumab	12 (0.8%)
**Treatment status**	
Active	620 (41.7%)
Discontinued	866 (58.3%)
Due to ineffectiveness	550 (37.0%)
Due to side-effects	203 (13.7%)
Other reasons	113 (7.6%)
**Biologic Naive**	
No	395 (27%)
Yes	1,091 (73%)

[1]n (%); Median (Q1, Q3)

The synthetic dataset was generated based on an existing cohort of patients with psoriasis treated with biologics to ensure that the simulated data closely reflected real-world clinical practice. The data were derived from an extraction of the prospective, multicenter, long-term Continuous Assessment of Psoriasis Use Registry with Biologics (BioCAPTURE) registry (https://www.biocapture.nl/) in June 2025. Since 2005, BioCAPTURE has systematically collected data on patients with psoriasis receiving biologics and currently includes data from four academic and nineteen non-academic centers across the Netherlands. The BioCAPTURE registry was approved by the relevant ethics committee. Although informed consent was not legally required for this non-interventional registry study under Dutch law, written informed consent was obtained from all registered patients.
All treatment episodes of etanercept, adalimumab, ustekinumab, secukinumab, ixekizumab, guselkumab, brodalumab, risankizumab, tildrakizumab, certolizumab and bimekizumab were included in the synthetic dataset. To facilitate methodological demonstrations and ensure that the statistical procedures could be illustrated without complications arising from incomplete observations, the synthetic dataset was constructed without missing data. Missing variables were imputed according to the observed distributions in the original cohort (e.g., 60% males versus 40% males, 30% with psoriatic arthritis versus 70% without psoriatic arthritis). For treatment discontinuations with unknown reasons, outcomes were assigned as either treatment ineffectiveness or adverse events. These assignments were made in proportions that allowed adequate demonstration of the competing risk methodology. Consequently, the resulting dataset preserves the overall characteristics and distributions of the original patient population while providing a complete dataset suitable for methodological illustration. As the dataset was supplemented for illustrative purposes, it should be emphasized that no substanstive conclusions can be drawn from the examples presented. Detailed characteristics of the final synthetic study population are presented in Supplemental Table S1.
